# Protection against Sexual Violence in the Colombian Legal Framework: Obstacles and Consequences for Women Victims

**DOI:** 10.3390/ijerph18084171

**Published:** 2021-04-15

**Authors:** Sandra M. Parra-Barrera, Nieves Moyano, Miguel Ángel Boldova, María del Mar Sánchez-Fuentes

**Affiliations:** 1 Human Rights and Sex Education Research Plan Foundation, Barranquilla 080001, Colombia; abogadasandraparra@gmail.com; 2 Faculty of Humanities and Science Education, University of Jaén, 23009 Jaén, Spain; mnmoyano@ujaen.es; 3 Department of Criminal Law, Philosophy of Law and History of Law, University of Zaragoza, 50009 Zaragoza, Spain; mboldova@unizar.es; 4 Department of Social Sciences, Faculty of Human and Social Sciences, University of la Costa, Barranquilla 080002, Colombia; 5 Department of Psychology and Sociology, Faculty of Human and Social Sciences, University of Zaragoza, 44003 Teruel, Spain

**Keywords:** sexual violence, regulatory framework, criminal legislation, rights, procedural guarantees

## Abstract

Sexual violence is a type of gender-based violence (GBV), as it is one of the different types of violence that is exerted against women. Sexual violence infringes fundamental human rights, and denies women’s dignity and self-determination, personal development, and well-being. Despite international treaties and a regulatory framework that legally protects Colombian women against sexual violence, it is necessary to know the effectiveness of this regulatory framework in Colombia. Therefore, the main objective of this research is to examine criminal legislation on crimes of sexual violence in Colombia with a dual purpose: first, to analyze procedural guarantees for women victims of sexual violence; second, to determine obstacles for victims of sexual violence in accordance with the legal framework. We used a legal interpretation method to perform an analysis and interpret the law. The results found that, although sexual violence is considered a type of crime, procedural guarantees are not effective as victims encounter serious obstacles with negative consequences, such as the violation of fundamental human rights.

## 1. Introduction

Gender-based violence (GBV) has been conceptualized as “any act of gender-based violence that results in, or is likely to result in, physical, sexual or psychological harm or suffering to women, including threats of such acts, coercion or arbitrary deprivation of liberty, whether occurring in public or in private life” [1]. GBV refers to unequal relationships between men and women and is a type of violence due to traditional gender roles and the patriarchal culture [2]. According to the United Nations [3] and to the Council of Europe Convention on preventing and combating violence against women and domestic violence [4], there are different types of GBV; e.g., physical, psychological, economic, sexual (female sexual mutilation, forced abortion and sterilization, forced marriage, harassment and rape). Furthermore, all these types of violence have been treated as a global problem for development and public health [5,6], which is why the United Nations has promoted both policies and programs to reduce GBV. However, gender inequality is still present and is, therefore, one of the Sustainable Development Goals of the United Nations 2030 Agenda to achieve equality between men and women [7].

This research focuses on sexual violence because, unlike other types of GBV such as gender or domestic violence [8], sexual violence is not specifically and legally recognized in Colombia. The lack of a specific law against sexual violence has consequences for women who are victims of sexual crimes; in this sense it is advocated for the inclusion of the gender perspective in the law, as well as in criminal law [9]. The absence of specificity in the norm leads to the lack of specific training in sexual violence for those who administer justice and are in contact with the victim in the legal process (e.g., police, health personnel, justice administration personnel) and the lack of courts specialized in sexual crimes, unlike, for example, gender-based violence in Spain [10]. Another consequence of the lack of specific regulations is the absence of specialized comprehensive assistance and the recognition of the right to reparation for victims of sexual violence. This leads to secondary victimization [11], in addition to posing a serious limitation for the recognition of the human rights of the victims [12]. However, as sexual violence is an international, regional, and national concern, efforts are made to eradicate it. Security Council resolutions on women underline the importance of eradicating sexual violence and of extending international peace and security, and in national jurisdictions [1]. 

Sexual violence has been defined as “any sexual act, attempt to obtain a sexual act, unwanted sexual comments or advances, or acts to traffic, or otherwise directed against a person’s sexuality using coercion, by any person regardless of their relationship to the victim, in any setting, including but not limited to home and work” [13]. Therefore, sexual violence involves different sexual acts, ranging from verbal harassment to forced penetration, as well as different types of coercion, ranging from pressure to physical force [14,15].

The sexual violence problem is reflected in available data. Globally, it is estimated that approximately 35% of women have suffered physical violence by their partner or sexual violence by other people [14]. Other data indicate that around 15 million women aged between 15 and 19 have been victims of forced sexual relations [16]. In Latin America and the Caribbean, a previous research concluded that between 5.8% and 13.4% of women report having been forced to have sexual relations [17].

In Colombia, the National Demographic and Health Survey [18] shows that 7.6% of women reported having suffered sexual violence by their partner. The survey results reveal that a higher proportion of women with a lower level of education and a lower socio-economic level reported having been victims of sexual violence by their partner. The same survey indicates that 4.5% of women declared having been victims of sexual violence by a person other than their partner. However, in 19.3% of the cases the aggressor was someone known to the victim (ex-partner, relative, acquaintance, stepfather, or father) and the aggressor was an unknown person in 10.8% of the cases. On sexual harassment prevalence (touching, feeling uncomfortable or intimidated on the street), 17.9% of women aged between 13 and 49 declared they had been touched without their consent, with these acts occurring more frequently at home (35.3%), followed by acts on the street (26.6), public transport (17.7%), somewhere else (16.8%) and education institution (4.5%). Other more recent data reported by the Institute of Legal Medicine [19], showed that 1987 girls and women have been victims of sexual violence. These data corresponded only to the months of January and July in 2018. The most recent data, and according to the Prosecutor’s Office [20], revealed that there had been at least 50,863 complaints of sexual crimes in 2020, in which women were victims of 28,600.

Sexual violence is a global problem that violates human rights and fundamental freedom, especially for women [21]. This type of violence denies women their dignity and self-determination [2,22], personal development and general well-being [23]. Women who are victims of sexual violence more frequently than women who have not suffered sexual assaults are diagnosed with sexually transmitted infections such as chlamydia, herpes, and genital warts [24], cardiovascular diseases [25], cancer [26], post-traumatic stress disorder [27], depression [28] and anxiety disorder [29]. Therefore, women who are victims of sexual violence are at greater risk of suffering physical and/or psychological health problems [30,31,32]. Furthermore, victims are also at risk of secondary victimization [33].

It is noteworthy that there are international treaties that apply laws to protect and guarantee human rights and fundamental freedom related to violence in general, and to violence against women in particular. The Convention on the Elimination of All Forms of Discrimination Against Women (CEDAW) [34] stands out, in which the States that ratify this Convention, such as Colombia [35], are obliged to promote effective legal protection of women’s rights. Another treaty is the Convention against Torture and Other Cruel, Inhuman or Degrading Treatment or Punishment [36], which is also ratified by Colombia [37]. Although it is not a specific international treaty against sexual violence, it is another instrument that forms part of the right to, and protection of, human rights and must, therefore, be taken into account when talking about sexual violence. Sexual violence of all types is an act that causes physical and/or mental discomfort [23,30,31,32] and is, therefore, considered cruel, inhuman, or degrading treatment for violating the right to equality and involving discriminatory acts [21].

It is also necessary to highlight the first specific international declaration on violence against women, the Declaration on the Elimination of Violence against Women (DEVAW) [1]. This Declaration recognized GBV as a violation of human rights and defined the violence against women concept by noting that it refers to any type of violence suffered by women that may entail damage, and physical, sexual, or psychological suffering. In addition, Article 4 establishes the need for States to condemn GBV and to develop the necessary measures to eliminate these types of violence, such as: preventing and punishing GBV; developing action plans to protect women; guaranteeing specialized assistance to victims; developing and applying specialized training in GBV for authorities, etc. [1].

Regionally, another treaty on GBV, ratified by Colombia [38], is the Inter-American Convention to Prevent, Punish and Eradicate Violence against Women [39]. This Convention also affirms that GBV implies the violation of women’s human rights and fundamental freedom by taking sexual violence as a type of violence against women that can be perpetrated or tolerated by the State (Art. 2). Like DEVAW [1], this Convention establishes that States must take the necessary measures to prevent, punish and eradicate violence, to develop fair and effective legal procedures, and to adopt judicial and administrative mechanisms to compensate victims. In addition, States must issue reports to specify the measures taken in accordance with the Convention’s provisions [39].

The above-cited Conventions play an essential role because, on the one hand, they recognize the existence of a specific problem and, on the other hand, they oblige States that ratify these Conventions to develop and implement the necessary measures to eradicate violence against women in all its forms. However, despite the existence of international and regional treaties on GBV, no previous research has examined the effectiveness of the regulatory framework that legally protects Colombian women against sexual violence. In addition, the numbers of victims of sexual violence are on the rise [18,19,20]. Therefore, the main objective of this pioneering research is to examine criminal legislation on crimes of sexual violence in Colombia. The specific objectives are to analyze the procedural guarantees for women victims of sexual violence and to determine the obstacles for victims of sexual violence in accordance with the legal framework.

## 2. Materials and Methods

### 2.1. Study Design

In the current research we employed the method of legal interpretation [40]. We use this method because, according to our objectives, it was our intention to carry out an analysis and interpret the law. The does not only refers to a system of legal precepts ready to be applied, but it goes further and implies interpretive social practice. This renders the legal interpretation method the most appropriate one to reveal contradictions and/or deficiencies in norms [40].

### 2.2. Materials

In this research, we analyzed the legal framework in relation to sexual violence in Colombia. Therefore, the criminal codes, the laws that have been modifying aspects related to crimes of sexual violence, were analyzed in order to analyze the punitive evolution of sexual crimes. In addition, we also analyzed laws that establish rights and guarantees for women victims of sexual violence. Finally, the protection judgments were also included, in which the Constitutional Court recognizes the violated rights of women victims of sexual violence, to examine the obstacles that women face in the judicial system. Table 1 shows the legal documents and contents analyzed.

### 2.3. Procedure

First, we analyzed the penal codes, decrees and laws that modified the articles related to crimes of sexual violence. Second, we sought the laws that establish rights and guarantees for women victims of sexual violence. These documents are public and can be consulted online. Finally, and through the website of the Constitutional Court of Colombia, we obtained the tutela judgments related to the violation of rights in cases of sexual violence in adult women. The above was carried out between the months of February and March 2021.

## 3. Results

### 3.1. Criminal Legislation on Crimes of Sexual Violence in Colombia

Crimes against sexual freedom in criminal law have undergone legislative changes over the years. In the Criminal Law of Colombia, three types of punitive conduct regarding sexual violence are contemplated: carnal access, violent sexual act, and abusive sexual acts, committed according to the following provisions: violently, with someone who cannot oppose and abusively in a minor aged under 14 years. Another factor in the normative evolution is the classification of sexual crimes. The Penal Codes of 1936 [41] and 1980 [42] establish the following types of crimes: carnal violence, rape, dishonest abuse and corruption of minors. However, this name was modified with Law 360 of 1997, in which sexual crimes were renamed crimes against sexual freedom and human dignity [43]. Table 2 shows the articles about crimes of sexual violence in the three Criminal Codes [41,42,44], as well as the laws that have reformed sanctions and punishable behaviors [43,45].

The Penal Code of 1936 established eight articles on crimes of sexual violence [41]: Article 317 referred to carnal access; Articles 318–321 established conditions for increased punitive penalty; Article 322 established reducing a penalty if the woman victim was a prostitute; Article 323 also declared annulling the penalty in the event of the offender marrying the victim (see Table 2).

The Penal Code of 1980 established 10 articles on punitive sexual violence acts [42]. For the first time, different types of criminal acts were declared: violent carnal access (Article 298), violent sexual act (Article 299), sexual act with a person that is not able to resist (Article 300), carnal access by deceit (Article 301), sexual act through deceit (Article 302), abusive carnal access with a minor aged under 14 years (Article 303), abusive carnal access with the inability to resist (Article 304) and corruption of minors (Article 305). The circumstances that led to increase the punishment were included (Article 306) and it was once again established, as set out in the Penal Code of 1936 [41], that there the punishment would be annulled in the event of the aggressor and victim marrying. Law 360 of 1997 [43] modified some of the articles established in the Penal Code of 1980 [42] that refer to crimes of sexual violence, particularly Articles 298, 299, 300, 303 and 305. This modification consisted of increasing punishment for offenders (see Table 2).

The Penal Code of 2000 included seven articles about crimes of sexual violence [44]. These seven articles considered previous crimes, namely: violent carnal access (Article 205), violent sexual act (Article 206), carnal access or sexual act with someone incapable of resisting (Article 207), carnal access (Article 208) or sexual act (Article 209) with a minor aged under 14 years, and carnal access or abusive sexual act and unable to resist (Article 210). In addition, there were two major differences in this Penal Code compared to previous ones [41,42] because it included a definition of carnal access (article 212) and, in addition, and an article about the punishment being annulled if the aggressor disappeared and if the victim married. Some of the articles on sexual violence in this Penal Code [44] were modified by Law 1236 of 2008 [45], by amending articles about crimes of sexual violence, particularly Articles 205, 206, 207, 208, 209, 210 and 211 of the Penal Code of 2000 [44]. The modification consisted in increasing the punishment for offenders (see Table 2).

### 3.2. Rights and Guarantees for Women Victims of Sexual Violence in Colombia

The first time that rights were established for victims of sexual violence in Colombia was by Law 360 in 1997 [43]. The established rights were the following: right to dignity, privacy and respect during interviews or actions for legal, medical or social assistance purposes; right to information on legal proceedings deriving from a punishable act; the right to information on available services to meet the needs generated by a crime; the right to free counselling by qualified personnel; the free right to the following services: examination and treatment to prevent STIs, including HIV/AIDS, examination and treatment for physical and emotional trauma; collection of legal medical evidence; information about the possibility of accessing compensation for damage caused by the criminal act. In addition, Article 16 declared the creation of Specialized Prosecution Units with their Technical Investigation Corps for crimes against sexual freedom and human dignity throughout the country. In all these units there will be a psychologist to advise officials during interviews with victims, as well as guidance for victims and them expressing their views to prosecutors [43].

Second, it is necessary to highlight Law 1257 of 2008, which is made up of eight chapters and 39 articles, and the purpose of which is to guarantee a life free of violence for women [8]. One important aspect of this law is the conceptualization of different types of violence against women (Arts. 2 and 3) and, although sexual violence is included, this law places more emphasis on domestic violence, or what may be considered gender violence; that is, violence committed by a victim’s partner or ex-partner. Of the rights established for victims, the following stand out: the right to receive comprehensive care, including legal advice and specialized legal technical assistance; the right to choose the sex of the professional who makes the medical-legal examinations; the right to receive specialized medical, psychological, psychiatric, and forensic care; the right to repair from, and guarantees of the non-repetition, of the acts constituting violence; the right to protection mechanisms (Arts. 7 and 8); the right to immediate protection (Art. 16). The approval of this norm symbolizes the formal recognition in Colombia of GBV, including sexual violence.

Third, it is worth highlighting Law 1719 of 2014, by means of which measures are adopted to guarantee victims of sexual violence access to justice, especially sexual violence during armed conflicts [46]. This regulation modified some articles of Law 599 of 2000 [44] and of Law 906 of 2004 [47] as a consequence of the international commitment assumed by the State of Colombia to guarantee human rights and prevent sexual violence.

Law 1719 of 2014 is made up of seven chapters and 32 articles [46], Table 3 shows the rights and procedural guarantees for the victims. Regarding the rights and guarantees of victims of sexual violence, the following rights are established: Intimacy and privacy; copy of denunciation and related documents such as medical examination; equality and non-discrimination by those who administer justice (non-discrimination based on their behavior, sexual orientation); right to care by people with training in human rights; the right to non-confrontation with the aggressor, to not submitting repetitive tests; right to care in safe places; right to be protected; right not to prejudge the victim; right to accompaniment and legal advice in all procedural stages; right to the consideration of special vulnerability; right to information and care about the possibility of abortion. In addition, this Law also includes other rights related to the investigation of the sexual crime, recommendations for judicial officials and the treatment of evidence, such as consent, which should not be inferred due if the victim lacks resistance; for example, the absence of traces of liquid and/or DNA may not be a sufficient reason to believe that the event did not occur.

The law also establishes protection measures for the main purpose of guaranteeing victims of sexual violence being able to access justice [46]. Of these measures, apart from guaranteeing those already established in Law 1257 of 2008, there is the possibility of psychosocial care for victims and access to victim protection programs is established. In addition, victims of sexual violence have the right to comprehensive free health care, including psychosocial care, with specialized professionals. Regarding repair measures, it is established that victims have the right to comprehensive repair, which must include restitution, compensation, satisfaction, and rehabilitation measures, and guarantees of non-repetition. Finally, another relevant factor is that which refers to the Monitoring Committee, created by Law 1257 of 2008 [8], to evaluate the obligations of institutions being fulfilled and to, thus, ensure the procedural guarantees and rights of the victims of sexual violence.

### 3.3. Obstacles for Women Victims of Sexual Violence at the Judicial Level in Colombia

Next, the three most recent Constitutional Court tutela judgments on sexual violence are analyzed to understand the obstacles faced by victims of sexual violence in judicial terms in Colombia.

Judgment T-211 of 2019 [48]. A woman was a victim of sexual violence; specifically, she suffered violent carnal access by paramilitaries in an event closely related to an armed conflict in August 2004. The victim filed a complaint, but she considered that the Administrative Unit for Attention and Comprehensive Reparation to Victims (UARIV) violated his rights. For this reason, in July 2018 the woman instituted tutelar action with which she requested the protection of her fundamental rights to due process, good faith, human dignity, rights of victims in armed conflicts and administrative repair. In Judgment T-211 of 2019, the Court ruled that the UARIV violated the rights manifested by the victim and granted the protection of her fundamental rights to become a recognized victim of armed conflict, due process, good faith, life in decent conditions and comprehensive repair. It particularly stated that the victim should be included in the Single Registry of Victims as a victim of crime against freedom and sexual integrity during an armed conflict to enjoy the benefits that result from it. Furthermore, the judgment notified that the UARIV should begin to provide psychological and psychosocial care by a gender differential approach to improve the victim’s psychological health [48].

Judgment T-126 of 2018 [49]. A woman was a victim of sexual violence and suffered sexual abuse and violent carnal access. These events occurred in July 2003. The victim filed a complaint, but she considered that the Criminal Chamber of the Superior Court of the Judicial District violated her rights by downplaying the importance of her statement. Thus, in May 2017, she filed constitutional action through which she requested the protection of her fundamental rights to human dignity, due process, an effective judicial remedy and guarantees of non-repetition. Regarding legal problems, in the second instance judgment issued during the ordinary process, the aforementioned rights were violated as revictimizing assertions were made that devalued the victim’s statements, and the required evidentiary assessment standards were ignored in the jurisprudence of matters in which sexual violence is investigated. It was specified that the Superior Court decision incurred several irregularities: a revictimizing approach in the sentence, ignoring regulations and jurisprudence regarding judicial evidence in cases of sexual violence; inconsequential contradictions to reduce the credibility of the victim’s statements; occurrence of fact; the Court’s analysis focused on identifying inconsistencies in the victim’s statements, rather than on fact; no evaluation of the probative material that demonstrated sexual violence. However in this judgment, the Constitutional Court ruled that the claims on the erroneous assessment of evidence in matters of sexual violence should be resolved first by an extraordinary appeal as it was the ideal mechanism [49].

Judgment T-418 of 2015 [50]. A woman was the victim of sexual violence when she suffered violent carnal access in 2010 by members of a group on the fringes of the law and was, therefore, an event related to the armed conflict. The victim instituted a tutela action in which she requested the protection of her fundamental rights. In this judgment, the Court declared the violation of rights and thus ordered the UARIV to design a plan and adopt measures for restitution, rehabilitation, satisfaction and guarantees of non-repetition. The plan included the provision of mental health services, and the provision of medical health services had to take a gender differential approach. It also ordered the Ministry of Health and Social Protection to undertake a plan to verify and monitor the implementation of what was ordered to the UARIV and the Ombudsman’s Office to monitor the orders issued in this judgment and the measures taken by the different entities in relation to the provision of mental health services [50].

After analyzing the guardianship judgments resolved by the Court, it was found that the women victims of sexual violence in Colombia encounter judicial-type obstacles, above all in relation to the violation of due process, life in dignified conditions and comprehensive repair. Thus, some obstacles were: a very long time elapsing between the application for protection being presented and effective protection coming into play; inadequate measures to protect the victim comprehensively (physical health, psychological health, specialized care, etc.) and the absence of a gender perspective.

## 4. Discussion

Sexual violence is one of the most egregious types of GBV and, therefore, violates women’s human rights and fundamental freedom. Although Colombia ratifies, on the one hand, international treaties with the force of law [35,37,38] and, on the other hand, a regulatory framework to legally protect women victims of sexual violence, it is essential to know the effectiveness of the regulatory framework in Colombia. Therefore, this research followed an analytical approach whose main objective was to examine criminal legislation on crimes of sexual violence in Colombia. In addition, the rights, procedural guarantees, and obstacles for women victims of sexual violence were analyzed. After these analyses, it was verified that criminal legislation has been submitted to modifications over the years, and rights and procedural guarantees are established in laws, but victims of sexual violence face various judicial-type obstacles which particularly entail revictimization.

After analyzing legislation on the structure of the criminal offenses and legal rights protected by the Penal Codes of 1936, 1980 and 2000 [41,42,44] on sexual violence, differences in normative evolution were observed. For example, active subjects and the description of carnal access itself have evolved, and undergone changes that are established in criminal law.

In the Penal Codes of 1936 [41] and 1980 [42], the influence of moral factors, religious prejudices, and patriarchal stereotypes as to the classification of sexual crimes were verified. An example of this was reducing by half a penalty if the victim of crimes was a prostitute woman (Art. 322) [41] and cancelling the criminal penalty if the aggressor married the woman victim (Art. 323) [41] and (Art. 307) [42]. Another aspect of normative evolution was that which referred to the classification of sexual crimes. In the Penal Codes of 1936 [41] and 1980 [42], the following types of crimes were set out: carnal violence, rape, dishonest abuse, and minor corruption. However, this name was modified with Law 360 of 1997, in which sexual crimes were renamed crimes against sexual freedom and human dignity [43].

Thus, it was found that the criminal law of sexual violence has changed since the promulgation of the 1991 Political Constitution [51]. One example of this was Law 360 of 1997 [43] and Law 1236 of 2008 [45] in which, on the one hand, the legal protected right against sexual crimes was established and, on the other hand, a punitive increase for some crimes was set out. In addition, for normative evolution regarding criminal types of sexual violence, there is a need for the legislator to eliminate from Penal Codes the crimes in which the protected legal right is, or, in fact, moral conceptions are, not precisely defined. The foregoing relies on the fact that the State of Colombia has ratified different international Treaties, such as the Inter-American Convention to Prevent, Punish and Eradicate Violence against Women [38] and, therefore, has the obligation to repeal the criminal provisions and laws that constitute discrimination against women.

The rights and procedural guarantees for women victims of violence have also evolved. It was not until Law 360 of 1997 was implemented [43] that rights were enshrined for victims of sexual violence in the criminal process for the first time. Subsequent modifications were made with Law 1257 of 2008 [8] and Law 1719 of 2014 [46]. In general, rights and procedural guarantees included: the right to dignity, privacy, confidentiality, and respect during actions for legal and medical purposes; the right to free guidance by qualified personnel; the right to medical, psychological, and specialized psychosocial treatment with a gender perspective; the right to comprehensive priority care; the right to repair and guarantees of non-repetition; the right to not be subjected to repetitive tests. The recognition of these rights and procedural guarantees are fundamental as they provide women with legal security.

Despite the rights and procedural guarantees established in the regulatory norms in Colombia, women victims of sexual violence face obstacles. These obstacles are related to structural or normative aspects and/or factors related to prejudices and stereotypes given the lack of specialization in GBV and sexual violence by those who administer justice. For example, one of the obstacles is the length of time the judicial process lasts [48], and even though victims of sexual violence in relation to the armed conflict have the right to urgently receive preferential treatment [40]. Another obstacle is the difficulties in practice for those who administer justice, for the registration in the single registry of victims [48] and therefore the difficulty in the access to routes of attention, assistance, and protection on the part of professionals with training specific in sexual violence. In other words, victims of sexual violence have difficulties in providing health care with a gender perspective [48,50]. Another obstacle is reducing the credibility of the victim by those who administer justice [49], thus producing secondary victimization. Finally, another major obstacle is the difficulty in obtaining guarantees of non-repetition.

The main obstacle is institutional violence at all stages from the very moment the victim files a complaint [52]. Consequently, this occurs with secondary victimization, which takes place when the legal system and other public institutions pay insufficient or inadequate attention, which entails a new violation of victims’ rights [21,53]. At the judicial level there are several aspects associated with secondary victimization, some of which are: prolongation of the trial, lack of privacy and protection, lack of information about the process, professionals’ subjectivity and/or lack of specialization, the classification of crimes and the victim’s credibility [54,55]. In addition, the tutela judgments resolved by the Constitutional Court show that women victims of sexual violence are revictimized by the judicial system, and women’s rights and procedural guarantees are violated by the system itself [48,49,50]. One of the limitations in justice that is related to the aforementioned obstacles is related to sound criticism. Indeed, the Supreme Court conceives rules of sound criticism referring to logic, maxims of experience or rules of science [49]. However, these rules do not clarify how to assess evidence, but they facilitate the presence of prejudices in sentences.

### Limitations

The current research has certain limitations. The first lies in the fact that since an analysis of the law and sentences was carried out, whose results cannot be generalized to all women victims of sexual violence in Colombia. Another limitation is that only the most recent guardianship judgments on sexual violence by the Constitutional Court were analyzed. For this reason, future research is recommended to systematically examine and analyze sentences on sexual violence in Colombia.

## 5. Conclusions

This study analyzes for the first time the Colombian criminal legislation regarding crimes of sexual violence with the purpose of examining the rights and procedural guarantees of the victims as well as the obstacles that the victims encounter at the judicial level. Therefore, this research is pioneering and will allow both legal professionals and academics to know the status regarding the legal framework of sexual violence, it may be useful to promote legal reform on crimes of sexual violence or go further and promote a law comprehensive on sexual freedom.

The International Conventions and Treaties on violence against women have been essential for the criminal law of the State of Colombia to advance after excluding and modifying criminal types with sexist and discriminatory overtones, as it has been possible to verify after carrying out the analysis of the punitive evolution of crimes of sexual violence. The legal framework contains substantial formal protection of guarantees and rights that victims of sexual violence have, although those who administer justice are unaware of, or do not apply, formal protection. This has been verified by analyzing the rights and procedural guarantees and by carrying out an analysis of the obstacles at the judicial level that victims of sexual violence encounter. Finally, it is important to highlight constitutional action; that is, tutela action seeks the protection of fundamental rights. In the analyzed judgments, the High Court ruled the protection of victims’ rights, which shows that the criminal judicial process is often ineffective in protecting rights and procedural guarantees for victims of sexual violence.

Considering the above, it is recommended the gender perspective and a new legal feminism trend to those who administer justice through workshops, seminars and reflections on the legal rights protected by crimes of sexual violence to guarantee the rights of victims and to avoid secondary revictimization as it represents the violation of a new right.

## Figures and Tables

**Table 1:** List of legal frameworks and documents.

Frameworks	Contents Analyzed
Law 95 of 1936	Articles related to sexual crimes.
Decree 100 of 1980	Articles related to sexual crimes.
Law 360 of 1997	Articles related to sexual crimes. Rights for women victims of sexual violence.
Law 599 of 2000	Articles related to sexual crimes.
Law 236 of 2008	Rights and procedural guarantees for women victims of sexual violence.
Law 1257 of 2008	Rights and procedural guarantees for women victims of sexual violence.
Law 1719 of 2014	Rights and procedural guarantees for women victims of sexual violence.
Judgment T-211 of 2019	Obstacles for women victims of sexual violence.
Judgment T-126 of 2018	Obstacles for women victims of sexual violence.
Judgment T-418 of 2015	Obstacles for women victims of sexual violence.

**Table 2:** Punitive evolution of sexual violence.

Penal Code	
Law 95 of 1936	Art. 317. Carnal access: anyone who subjects another person to carnal access, without their consent and by means of physical or moral violence. Penalty: 2–8 years in prison. Article 318. Increasing the penalty by one quarter in the following cases: 1st If the crime is committed with someone who is a virgin or of irreproachable honesty. 2nd If it is committed with the help of another person or other persons. 3rd If the responsible person has any character or post in charge that confers him/her particular authority over the victim or prompts her to place her trust in him. Penalty: increased by one quarter. Art. 319. Increased penalty if the acts carried out on victims cause their death or seriously damage their health. Penalty: 3–12 years in prison. Art. 320. Whoever gains carnal access to a woman over the age of 14 years by deceptive maneuvers or trickery of any kind, or by seducing her by a formal promise of marriage; or carnal access with someone who suffers mental alienation or is in a state of unconsciousness. Penalty: 1–6 years in prison. Art. 321. The cases provided for in Article 318 and in that of venereal contamination. Penalty: increased by one quarter. Art. 322. The penalties indicated in the previous chapters will be reduced by up to one half if the victim of the crimes provided therein is a prostitute or a public woman. In this case, one cannot proceed except by virtue of private accusation. Art. 323. The person responsible for the crimes mentioned in the two previous chapters shall be exempt of punishment if he marries the offended woman. Art. 324. Anyone who performs an erotic-sexual act on the body of someone age aged over 16 years, other than carnal access by using any of the means provided in Articles 319 and 322. They shall incur the same penalty. Whoever consumes homosexual carnal access, whatever their age. Penalty: 6 months to 2 years in prison.
Decree 100 of 1980	Art. 298. Violent carnal access. Whoever performs carnal access with another person through violence. Penalty: 2–8 years in prison. Art. 299. Violent sexual act. Anyone who performs a sexual act other than carnal access on someone else person through violence. Penalty: 1–3 years in prison.
	Art. 300. Sexual act with a person rendered incapable of resisting. Anyone who performs carnal access with someone who is in a situation of being unable to resist or is in a state of unconsciousness or is in a condition of psychic inferiority that prevents him/her from understanding the sexual relation. Penalty: 2–8 years in prison. Penalty: 1–3 years in prison if a sexual act other than carnal access is performed. Art. 301. Carnal access through deceit. Anyone who through deception obtains carnal access with a person aged over 14 and under 18 years. Penalty: 1–5 years in prison. Art. 302. Sexual act through deceit. Anyone who by deception performs a sexual act other than carnal access on someone aged over 14 and under 18 years. Penalty: 6 months to 2 years in prison. Art. 303. Abusive carnal access with a minor aged under 14 years. Whoever carnally accesses a person aged under 14 years. Penalty: 1–6 years in prison. Art. 304. Abusive carnal access and unable to resist. Whoever carnally accesses a person in a state of unconsciousness, suffers from a mental disorder or is unable to resist. Penalty: 2–6 years in prison. Penalty: 1–3 years in prison if access is not carried out, but various sexual acts are. Art. 305. Corruption. Whoever performs various sexual acts of carnal access with someone aged under 14 years, or in their presence, or induces them to sexual practices. Penalty: 1–4 years in prison. Art. 306. Circumstances of punitive aggravation, increased from one third to one half, in the following cases: 1. If it is committed with the assistance of someone else or other persons. 2. If the responsible person has any character, position or position that confers him/her particular authority over the victim or prompts her to place her trust in him. 3. If the victim becomes pregnant. 4. If venereal contamination occurs. 5. If the crime is carried out on a person aged under 10 years. Art. 307. Termination of the criminal action for marriage. If any of the authors or participants in the crimes described in the previous article’s contracts valid marriage with the taxpayer, the criminal action for them all shall be annulled.
Law 360 of 1997	Art. 298. Violent carnal access. Whoever has carnal access with someone else through violence. Penalty: 8–20 years in prison. Whoever has carnal access with someone aged under 12 through violence. Penalty: 20–40 years in prison. Art. 299. Violent sexual act. Anyone who performs a sexual act other than carnal access through violence on another person. Penalty: 4–8 years in prison. Art. 300. Sexual act in persons rendered incapable of resisting. Whoever performs carnal access with someone who is unable to resist, is in a state of unconsciousness, or is in a condition of mental inferiority that prevents them from understanding the sexual relation or giving their consent. Penalty: 4–10 years in prison. Penalty: 2–4 years if a sexual act other than carnal access is performed. Art. 303. Abusive carnal access with a minor. Whoever carnally accesses a person under the age of 14. Penalty: 4–10 years in prison. Art. 304. Abusive carnal access and unable to resist. Whoever carnally accesses someone in a state of unconsciousness, who suffers from a mental disorder or is unable to resist. Penalty: 3–10 years in prison. Penalty: 2–4 years in prison if access is not performed, but other sexual acts are. Art. 305. Sexual acts with a minor under the age of 14. Whoever performs various sexual acts of carnal access with someone under the age of 14, in their presence, or induces them to sexual practices. Penalty: 2–5 years in prison.
Law 599 of 2000	Art. 205. Violent carnal access. Whoever performs carnal access with another person through violence. Penalty: 8–15 years in prison. Art. 206. Violent sexual act. Anyone who performs a sexual act other than carnal access through violence with another person. Penalty: 3–6 years in prison. Art. 207. Carnal access or sexual act in someone rendered incapable of resisting. Whoever performs carnal access with someone who is unable to resist, is in a state of unconsciousness or in psychic inferiority conditions that prevent him/her from understanding. Penalty: 8–15 years in prison. Penalty: 3–6 years in prison if a sexual act other than carnal access is performed. Art. 208. Abusive carnal access with a minor aged under 14 years. Whoever carnally accesses a person aged under 14. Penalty: 4–8 years in prison. Art. 209. Sexual acts with a minor aged under 14. Whoever performs various sexual acts of carnal access with a person aged under 14, or in their presence, or induces them to sexual practices. Penalty: 3–5 years in prison. Article 210. Carnal access or abusive sexual act and unable to resist. Whoever carnally accesses a person in a state of unconsciousness, who suffers from a mental disorder or is unable to resist. Penalty: 4–8 years in prison. Art. 212. Carnal access. For the purposes of the behaviors described in the previous chapters, carnal access means penetration of the penis anally, vaginally, or orally, as well as vaginal or anal penetration of any other part of the human body or another object.
Law 1236 of 2008	Art. 205. Violent carnal access. Whoever performs carnal access with another person through violence. Penalty: 12–20 years in prison. Art. 206. Violent sexual act. Anyone who performs a sexual act other than carnal access through violence on another person. Penalty: 8–16 years in prison. Art. 207. Carnal access or sexual act in a person rendered incapable of resisting. Whoever performs carnal access with someone who is unable to resist, is in a state of unconsciousness or in a condition of mental inferiority that prevents him/her from understanding the sexual relation or giving his/her consent. Penalty: 12–20 years in prison. Penalty: 8–16 years in prison if a sexual act other than carnal access is performed. Art. 208. Abusive carnal access with a minor aged under 14 years. Whoever carnally accesses a person aged under 14. Penalty: 12–20 years in prison. Art. 209. Sexual acts with a minor aged under 14 years. Whoever performs various sexual acts of carnal access with a person aged under 14, or in their presence, or induces them to sexual practices. Penalty: 9–13 years in prison. Article 210. Carnal access or abusive sexual act and incapable of resisting. Whoever carnally accesses a person in a state of unconsciousness, who suffers from a mental disorder or is unable to resist. Penalty: 12–20 years in prison. Penalty from 8–16 years in prison if access is not performed, but various sexual acts are. Art 211. Circumstances of punitive aggravation. The penalties for the crimes described in the previous articles shall be increased from one third to one half when: 1. conduct is committed with the assistance of another or other persons. 2. The person responsible has any character, position or position that confers him/her particular authority over the victim or prompts her to place her trust in him. 3. Contamination from sexually transmitted disease (STDs) occurs. 4. Carried out on a person aged under 14 years. 5. Carried out on a spouse or on the person whom one cohabits or has cohabited, or with the person with whom a child has been procreated. 6. Pregnancy occurs. 7. When the victim is an elderly person, or is physically, sensory, or mental handicapped.

**Table 3:** Procedural Rights and Guarantees of Law 1719 of 2014.

Law	Articles
Law 1719 of 2014	Art. 13. Right to intimacy, privacy, confidentiality; non-discrimination to specialized legal and psychological care; the right to not be subjected to repetitive tests. Art. 14. Investigations into crimes of sexual violence connected to armed conflicts shall be carried out. Art. 17. Obligation to conduct investigations once judicial officials have knowledge of the facts. Art. 18. Recommendations for judicial officials and treatment of evidence. Art. 19. Recommendations for the conduct of the investigation and assessment of the evidence. Art. 20. Crimes of sexual violence may not be investigated through the military criminal jurisdiction. Art. 21. Existence of technical-legal committees of the National Office of the Attorney General to investigate cases of sexual violence. Art. 22. Psychosocial care for victims and access to victim protection programs. Art. 23–24. Right to comprehensive free health care, including psychosocial care, with specialized professionals for the care of sexual violence. Art. 25. Right to comprehensive repair. Art. 26. Right to participate in defining repair measures. Art. 27–28. Establishment special rules for processing the incident of integral repair in cases of sexual violence connected to armed conflict. Art. 32. Monitoring Committee to evaluate the obligations of institutions being fulfilled.

## Data Availability

Not applicable.

## References

[1] United Nations General Assembly (1993). Declaration of the Elimination of Violence against Women.

[2] Gil M. (2015). Sexual violence as an attack against the dignity of women. RDUNED.

[3] United Nations General Assembly (1995). In Proceedings of the Report of the Fourth World Conference on Women.

[4] Council of Europe In Proceedings of the Council of Europe Convention on Preventing and Combating Violence against Women and Domestic Violence.

[5] Basile K.C., Smith S.G. (2011). Sexual violence victimization of women: Prevalence, characteristics, and the role of public health and prevention. Am. J. Lifestyle Med..

[6] Fernández-Fuertes A.A., Fernández-Rouco N., Lázaro-Visa S., Gómez-Pérez E. (2020). Myths about sexual aggression, sexual assertiveness and sexual violence in adolescent romantic relationships. Int. J. Environ. Res. Public Health.

[7] United Nations General Assembly (2015). Sustainable Development Goals (SDGs), Transform Our World: The 2030.

[8] Diario Oficial 47.193 Law 1257, from December 4th 2008 Whereby Norms of Awareness, Prevention and Punishment of Forms of Violence and Discrimination against Women are Issued, the Penal Codes, Penal Procedure, Law 294 of 1996 are Reformed and Other Provisions are Issued. https://www.oas.org/dil/esp/LAW_1257_DE_2008_Colombia.pdf.

[9] Monge-Fernández A., Bosch J.M. (2020). Women and Criminal Law: Need for Reform from a Gender Perspective?.

[10] Boletín Oficial del Estado 313 Organic Law 1/2004, of December 28, on Comprehensive Protection Measures against Gender Violence. https://www.boe.es/buscar/act.php?id=BOE-A-2004-21760.

[11] Faraldo P., Avale M. (2018). The Woolf. A before and after in the Regulation of Sexual Crimes in Spain.

[12] Miller A.M. (2004). Sexuality, violence against women, and human rights: Women make demands and ladies get protection. Health Hum. Rights.

[13] World Health Organization (2014). Violence against Women: Intimate Partner Violence and Sexual Violence against Women Intimate Partner and Sexual Violence have Serious Short- and Long-Term Physical, Mental and Sexual and Reproductive Health Problems for Survivors: Fact Sheet. https://apps.who.int/iris/bitstream/handle/10665/112325/WHO_RHR_14.11_eng.pdf?sequence=1&isAllowed=y.

[14] Struckman-Johnson C., Struckman-Jhonson D., Anderson P.B. (2003). Tactics of sexual coercion: When men and women won’t take no for an answer. J. Sex Res..

[15] World Health Organization (2013). Global and Regional Estimates of Violence against Women: Prevalence and Health Effects of Intimate Partner Violence and Non-Partner Sexual Violence. https://apps.who.int/iris/bitstream/handle/10665/85239/9789241564625_eng.pdf?sequence=1.

[16] UNICEF (2017). A Familiar Face: Violence in the Lives of Children and Adolescents. https://data.unicef.org/resources/a-familiar-face/#.

[17] Bott S., Guedes A., Goodwin M., Mendoza J.A. (2012). Violence against Women in Latin America and the Caribbean: A Comparative Analysis of Population-Based Data from 12 Countries.

[18] (2015). National Survey of Demography and Health, Executive Summary: Colombia. https://www.dhsprogram.com/pubs/pdf/FR334/FR334.pdf.

[19] Institute of Legal Medicine (2018). Data for Life.

[20] Office of the Attorney General of the Nation (2020). Accusatory Oral Penal System.

[21] Parra-Barrera S.M., Sánchez-Fuentes M.M., Panarese P., Martínez N., Suárez J.C. (2020). Legal framework of sexual violence: Sexual consent and risk of secondary victimization in Colombia. Cartography of Micromachisms: Dynamics and Symbolic Violence.

[22] Bogen K.W., Bleiweiss K., Orchowski L.M. (2019). Sexual violence is# NotOkay: Social reactions to disclosures of sexual victimization on twitter. Psychol. Violence.

[23] Sigurdardottir S., Halldorsdottir S. (2021). Persistent Suffering: The Serious Consequences of Sexual Violence against Women and Girls, Their Search for Inner Healing and the Significance of the# MeToo Movement. Int. J. Environ. Res. Public Health.

[24] Brookmeyer K.A., Beltran O., Abad N. (2017). Understanding the effects of forced sex on sexually transmitted disease acquisition and sexually transmitted disease care: Findings from the national survey of family growth (2011–2013). Sex. Transm. Dis..

[25] El-Serag R., Thurston R.C. (2020). Matters of the heart and mind: Interpersonal violence and cardiovascular disease in women. Am. Heart J..

[26] Gonzalez J.M., Jetelina K.K., Olague S., Wondrack J.G. (2018). Violence against women increases cancer diagnoses: Results from a meta-analytic review. Prev. Med..

[27] Cook J.M., Dinnen S., O’Donnell C. (2011). Older women survivors of physical and sexual violence: A systematic review of the quantitative literature. J. Women’s Health.

[28] Schnittker J. (2019). Sexual violence and major depression among women: Evidence for reciprocal relationships. Soc. Curr..

[29] Chandan J.S., Thomas T., Bradbury-Jones C., Russell R., Bandyopadhyay S., Nirantharakumar K., Taylor J. (2020). Female survivors of intimate partner violence and risk of depression, anxiety and serious mental illness. Brit. J. Psychiatry.

[30] Oram S., Khalifeh H., Howard L.M. (2017). Violence against women and mental health. Lancet Psychiatry.

[31] Bows H. (2018). Sexual violence against older people: A review of the empirical literature. Trauma Violence Abus..

[32] Ba I., Bhopal R.S. (2017). Physical, mental and social consequences in civilians who have experienced war-related sexual violence: A systematic review (1981–2014). Public Health.

[33] Walker H.E., Freud J.S., Ellis R.A., Fraine S.M., Wilson L.C. (2019). The prevalence of sexual revictimization: A meta-analytic review. Trauma Violence Abus..

[34] United Nations General Assembly (1979). Convention on the Elimination of All Forms of Discrimination against Women.

[35] Diario Oficial 35794 Law 51, June 2nd 1981, through Which the Convention on the Elimination of All Forms of Discrimination against Women is Approved. http://www.suin-juriscol.gov.co/viewDocument.asp?ruta=Lawes/1605470.

[36] United Nations General Assembly (1984). Convention against Torture and Other Cruel, Inhuman or Degrading Treatment or Punishment.

[37] Diario Oficial 37737 Law 70, from December 15 from 1986 by which the Convention against Torture and Other Cruel, Inhuman or Degrading Treatment or Punishment is approved. http://www.suin-juriscol.gov.co/viewDocument.asp?ruta=Lawes/1620301.

[38] Diario Oficial 42171 Law 248, from December 29 1995, through which the Inter-American Convention of Belém do Pará to Prevent, Punish and Eradicate Violence against Women is Ratified. https://www.defensoria.gov.co/public/Normograma%202013_html/Normas/Law_248_1995.pdf.

[39] Organization of American States (1994). A-61: Inter-American Convention to Prevent, Punish and Eradicate Violence against Women.

[40] Feteris E.T. (2017). Legal Argumentation and Legal Interpretation. Fundamentals of Legal Argumentation.

[41] Diario Oficial 23316 Law 95, from April 24 1936, Penal Code. https://www.suin-juriscol.gov.co/viewDocument.asp?id=1791348.

[42] Diario Oficial 35461 Decree—Law 100 from 23 1980, from 23 1980, Penal Code. https://normograma.info/men/docs/pdf/codigo_penal_1980.pdf.

[43] Law 360 from 1997, from February 7 1997, by Means of Which Some Rules of Title XI of Book II of the Decree-Law 100 from 1980 (Penal Code), Relative to Crimes against Sexual Freedom and Modesty, and Article 417 of Decree 2700 of 1991 (Criminal Procedure Code) is Added and Other Provisions are Issued. https://oig.cepal.org/sites/default/files/1997_col_Law360.pdf.

[44] Diario Oficial 44097 Law 599 from 2000, from August 31 2004, Penal Code. http://www.oas.org/juridico/spanish/mesicic2_col_Law_599_2000.pdf.

[45] Diario Oficial 47059 Law 1236 from 2008, from July 23rd 2008, by Means of Which Some Articles of the Penal Code related to Crimes of Violence are Modified. http://www.oas.org/dil/esp/Law_1236_de_2008_Colombia.pdf.

[46] Diario Oficial 49186 Law 1719, from June 18 2014, by Which Some Articles of Laws 599 of 2000, 906 of 2004 are Modified and Measures are Adopted to Guarantee Access to Justice for Victims of Sexual Violence, Especially Sexual Violence on the Occasion of the Armed Conflict, and Other Provisions. http://www.suin.gov.co/viewDocument.asp?ruta=Lawes/1687214.

[47] Diario Oficial 45658 Law 906 de 2004, from August 31 2004, Code of Criminal Procedure. http://www.suin-juriscol.gov.co/viewDocument.asp?ruta=Lawes/1670249.

[48] Judgment of Guardianship of the Constitutional Court of May 20, 2019, Judgment T-211/2019. https://www.corteconstitucional.gov.co/relatoria/2019/T-211-19.htm#:~:text=T%2D211%2D19%20Corte%20Constitucional%20de%20Colombia&text=Referencia%3A%20Expediente%20T%2D7.069.,a%20las%20V%C3%ADctimas%20%E2%80%93%20UARIV%2D.&text=Bogot%C3%A1%2C%20veinte%20(20)%20de,dos%20mil%20diecinueve%20(2019).

[49] Judgment of Guardianship of the Constitutional Court of May 9, 2018, Judgment T-126 de 2018. https://www.corteconstitucional.gov.co/relatoria/2018/t-126-18.htm#:~:text=T%2D126%2D18%20Corte%20Constitucional%20de%20Colombia&text=Tratados%20internacionales%20de%20derechos%20humanos,la%20violencia%20contra%20la%20mujer.

[50] Judgment of Guardianship of the Constitutional Court of July 3 2015, Judgment T-418 de 2015. https://www.corteconstitucional.gov.co/relatoria/2015/T-418-15.htm#:~:text=T%2D418%2D15%20Corte%20Constitucional%20de%20Colombia&text=Las%20personas%20que%20hayan%20sufrido,de%20su%20condici%C3%B3n%20de%20v%C3%ADctimas.

[51] Political Constitution (1991). Political Constitution from Colombia.

[52] International Amnesty I do not Consent. Stop Obstacles for Victims of Sexual Violence. https://www.es.amnesty.org/en-que-estamos/campanas/violencia-sexual-2018/.

[53] Baca E., Echeburúa E., Tamarit J.M. (2006). Handbook of Criminology.

[54] Albertin P., Soria M., Saíz D. (2006). Psychology of criminal victimization. Criminal Psychology.

[55] González J. (2006). The justification of the sentences and sound criticism. Rev. Chil. Derecho.

